# Oral hygiene, dentition, sexual habits and risk of oral cancer

**DOI:** 10.1054/bjoc.2000.1398

**Published:** 2000-11

**Authors:** R Talamini, S Vaccarella, F Barbone, A Tavani, C La Vecchia, R Herrero, N Muñoz, S Franceschi

**Affiliations:** Servizio di Epidemiologia, Centro di Riferimento Oncologico, via Pedemontana Occ., Aviano, 33081, Italy; Cattedra di Igiene, Dipartimento di Patologia e Medicina Sperimentale e Clinica, University of Udine, via Colugna, 40, Udine, 33100, Italy; Department of Epidemiology and International Health, School of Public Health, University of Alabama at Birmingham, 1665 University Blvd. #230J, Birmingham, AL, 35294-0022, USA; Istituto di Ricerche Farmacologiche “Mario Negri”, via Eritrea, 62, Milan, 20157, Italy; Istituto di Statistica Medica e Biometria, University of Milan, via Venezian, 1, Milan, 20133, Italy; Caja Costarricense de Seguro Social, San Jose, Costa Rica; International Agency for Research on Cancer, 150 cours Albert-Thomas, Lyon Cedex 08, 69732, France

**Keywords:** oral cancer, dentition, oral hygiene, sexual practices, oral sex, warts

## Abstract

In an Italian case-control study of oral cancer, number of missing teeth and other aspects of dental care were similar, but the general condition of the mouth, as indicated by gum bleeding, tartar deposits and mucosal irritation, was worse among oral cancer cases than controls. No differences were detected in sexual practices (including oral sex) and (previous) sexually transmitted infections. © 2000 Cancer Research Campaign


*British Journal of Cancer* (2000) **83**(9), 1238–1242
© 2000 Cancer Research Campaign
doi: 10.1054/ bjoc.2000.1398, available online at http://www.idealibrary.com on IDEAL®

Short Communication

### Oral hygiene, dentition, sexual habits and risk of oral cancer

**R Talamini[1], S Vaccarella[1], F Barbone[2,3], A Tavani[4], C La Vecchia[4,5], R Herrero[6,7], N Muñoz[7] and S Franceschi[1,7]**

[1]Servizio di Epidemiologia, Centro di Riferimento Oncologico, via Pedemontana, Occ., 33081 Aviano, Italy; [2]Cattedra di Igiene, Dipartimento di Patologia e Medicina Sperimentale e Clinica, University of Udine, via Colugna, 40, 33100 Udine, Italy; Department of Epidemiology and International Health, School of Public Health, University of Alabama at Birmingham, 1665 University Blvd. #230J, Birmingham, AL, 35294-0022, USA; [4]Istituto di Ricerche Farmacologiche "Mario Negri", via Eritrea, 62, 20157 Milan, Italy; [5]Istituto di Statistica Medica e Biometria, University of Milan, via Venezian, 1, 20133 Milan, Italy; [6]Caja Costarricense de Seguro Social, San Jose, Costa Rica; [7]International Agency for Research on Cancer, 150 cours Albert-Thomas, 69732 Lyon Cedex 08, France

**Summary** In an Italian case-control study of oral cancer, number of missing teeth and other aspects of dental care were similar, but the general condition of the mouth, as indicated by gum bleeding, tartar deposits and mucosal irritation, was worse among oral cancer cases than controls. No differences were detected in sexual practices (including oral sex) and (previous) sexually transmitted infections. © 2000 Cancer Research Campaign

**Keywords:** oral cancer; dentition; oral hygiene; sexual practices; oral sex; warts

In developed countries, over 80% of cancers of the oral cavity and pharynx are attributable to tobacco smoking and heavy alcohol consumption, with low intake of fresh fruit and vegetables playing a smaller role (Negri et al, 1993; Franceschi et al, 1999).

Since the earliest case-control studies on oral cancer (Wynder et al, 1957; Graham et al, 1977), poor oral hygiene (Franco et al, 1989), poor dentition (Zheng et al, 1990), missing teeth or denture use (Marshall et al, 1992) have been postulated as risk factors. Viruses, most notably human papillomavirus (HPV) (Franceschi et al, 1996), are suspected to have a role. A relation between oral cancer and sexual habits may also exist, but information is limited (Maden et al, 1992; Schwartz et al, 1998).

The present Italian case-control study was part of an international study on oral cancer and HPV carried out also in Spain, Northern Ireland, Poland, Cuba, Canada, India, Sudan and Australia, and coordinated by the International Agency for Research on Cancer (Herrero et al, 2000). Between July 1996 and June 1999 first incident cases of cancer of the oral cavity and oro-pharynx were identified in three hospitals of Friuli-Venezia Giulia region, North-East Italy. Eight percent of potential cases refused or were unable to participate. A total of 132 cases (median age = 60; range 27–86 years), including 33 women, were enrolled, distributed by subsite as: tongue, 54; mouth, 34; oro-pharynx, 39; unspecified, 5. All cases had their interview and oral examination before any cancer treatment. Controls were 148 subjects (median age = 60; range 30–83 years), including 45 women. They had been admitted to major hospitals in the study region for acute conditions unrelated to smoking or drinking habits (37% for acute surgical diseases; 18% for non-traumatic orthopaedic conditions; 18% for skin diseases; 12% for trauma; 11% for ear diseases; and 4% for eye diseases). Control subjects were frequency-matched with cases by age and gender. Four percent of contacted controls refused to participate.

Cases and controls were interviewed by trained nurses. Study subjects had given written consent to be included in the study and provide samples of buccal exfoliated cells, blood and (cases only) tissue biopsies. Questionnaire included information on socio-demographic characteristics, detailed histories of smoking and drinking habits, prior occurrence of sexually transmitted and other infections, cancer family history, and a 25-item dietary questionnaire. Indicators of oral hygiene were self-reported by means of 9 specific questions. The number of missing teeth which had not been replaced and the general oral condition, on the basis of presence of tartar, decayed teeth, and mucosal irritation, were evaluated by interviewers through inspection of the mouth. Interviewers were aware of case/control status.

Odds ratios (ORs) and corresponding 95% confidence intervals (CIs) were computed using unconditional multiple logistic regression models. All models included terms for age quinquennium and gender. Second-level models included also terms for smoking and drinking habits and intake of fruit and vegetables.

Cases and controls reported similar educational attainment (Table 1) and occupation (not shown). Conversely, cases showed a marked excess of heavy smokers (OR for ≥25 cigs/day versus never smokers = 14.8; 95% CI: 3.1–70.4) and heavy alcohol drinkers (OR for ≥ 84 drinks/week versus abstainers = 12.7; 95% CI: 2.8–56.4). Ex-smokers had a 2.4-fold risk of oral cancer compared to never smokers.

Cases reported that they brushed their teeth less often than controls, but the inverse association was substantially lowered by allowance for smoking and drinking habits and fruit and vegetable intake (OR = 1.4 for < 1 vs. ≥ 2 per day; 95% CI: 0.6–3.3) (Table 2). More cases than controls reported that their gums bled sometimes (OR = 1.8; 95% CI: 0.9–3.6) or always or nearly always

*Received 3 May 2000*
*Revised 21 June 2000*
*Accepted June 2000*

*Correspondence to:* S Franceschi

**Table 1** Odds ratios (OR) and corresponding 95% confidence interval (CI)[1] for cancer of the oral cavity and oro-pharynx according to education and smoking and drinking habits.[2] North-eastern Italy, 1996–1999

| | No. Cases | No. Controls | OR | (95% CI) |
|---|---|---|---|---|
| Education (years) | | | | |
| ≥ 9 | 35 | 43 | 1[3] | |
| 6–8 | 43 | 53 | 0.8 | (0.4–1.6) |
| ≤ 5 | 54 | 52 | 0.7 | (0.3–1.6) |
| $\chi^2_1$ for trend | 0.55; $P$ = 0.46 | | | |
| Smoking habit | | | | |
| Never smokers | 17 | 56 | 1[3] | |
| Ex smokers | 30 | 40 | 2.4 | (1.0–5.8) |
| Current smokers (cigs./day) | | | | |
| 1–14 | 25 | 27 | 2.4 | (1.0–5.9) |
| 15–24 | 41 | 22 | 5.7 | (2.2–15.0) |
| ≥ 25[4] | 18 | 3 | 14.8 | (3.1–70.4) |
| $\chi^2_1$ for trend | 16.08; $P$ < 0.001 | | | |
| Drinking habit (drinks/week)[5] | | | | |
| Abstainers | 8 | 16 | 1[3] | |
| 1–20 | 39 | 90 | 1.2 | (0.4–3.5) |
| 21–34 | 20 | 20 | 2.8 | (0.8–10.0) |
| 35–55 | 24 | 9 | 7.2 | (1.8–28.2) |
| 56–83 | 12 | 6 | 4.3 | (1.0–18.9) |
| ≥ 84 | 28 | 4 | 12.7 | (2.8–56.4) |
| $\chi^2_1$ for trend | | | 22.33; $P$ < 0.001 | |

[1] Estimates from unconditional logistic regression equations, including terms for gender, age, fruit and vegetable intake and smoking and drinking habits. [2] Some strata do not add up to the total because of missing values. [3]Reference category. [4] Cigar and pipe smokers were included in this category. [5] One drink corresponds to approximately 125 ml of wine, 330 ml of beer, and 30 ml of liquor (i.e., about 12 g of ethanol).

(OR = 3.9; 95% CI: 1.2–12.6). Mouthwash use was infrequent and did not seem to influence oral cancer risk. Similar proportions of cases (45%) and controls (47%) reported wearing a removable denture. Neither ever nor long-duration (≥ 10 years) use of denture was associated with an increased risk. Lack of dental check-ups in the last 20 years was not associated with risk after allowance for major risk correlates (OR = 1.1; 95% CI: 0.5–2.6). At visual inspection of the mouth, the number of missing teeth was not significantly associated to oral cancer risk (OR = 1.4; 95% CI: 0.6–3.1), whereas a poor general oral condition was 4.5-fold (95% CI: 1.8–10.9) more frequent among cases than control subjects, even after allowance for smoking and drinking habits and fruit and vegetable intake (Table 2).

Cases and controls reported similar previous frequency of warts at various body sites, and oral or genital herpetic lesions (Table 3). An excess of borderline statistical significance emerged for candidiasis of the mouth, but not of other sites (mostly genital). Three cases and one control reported syphilis, whereas for gonorrhoea the corresponding figures were three and four, respectively. No trend in oral cancer risk was found in relation to number of lifetime sexual partners (OR for ≥ 11 versus 1 partner = 1.6) or of partners who were prostitutes (OR ≥ 6 prostitute, versus none = 1.0, men only). Occasional (OR = 0.7) or frequent (OR = 1.3) practice of oral sex, and homosexual intercourse (OR = 1.0, men only) did not seem to affect oral cancer risk (Table 3). All variables were re-examined separately for cancers of the tongue, mouth and oropharynx, and among non-smokers, yielding similar risk patterns.

Poor oral hygiene has been postulated as a risk factor for oral cancer in several studies (Franco et al, 1989; Schildt et al, 1998) including a few where smoking and drinking habits were allowed for (Elwood et al, 1984; Zheng et al, 1990; Bundgaard et al, 1995; Velly et al, 1998). People who brushed their teeth infrequently generally showed RR in the 1.5–2.0 range, but the association was generally not significant, as in our present study. In a Chinese case-control study (Zheng et al, 1990) a 7-fold elevated risk emerged in men who reported they never brushed their teeth.

An association of oral cancer risk with number of missing teeth has been found in some (Zheng et al, 1990; Marshall et al, 1992; Bundgaart et al, 1995), but not all studies (Kabat et al, 1989). However, number of missing and not replaced teeth is strongly correlated with dental check-ups which, in turn, are inversely associated with oral cancer (Bundgaard et al, 1995).

Our study suggested that, albeit indicators of dental care and dentition were only marginally worse among cases, cases might have a poorer general mouth condition than control subjects, as indicated by more frequent gum bleeding and, at oral inspection, excesses of tartar deposits, decayed teeth and mucosal irritation. Such differences were similar for cancers of the mouth, tongue, and oro-pharynx, and they were confirmed among the few never smokers (ORs for frequent gum bleeding = 1.7, and for poor general oral condition = 2.9). Findings from oral inspection, however, must be interpreted with great caution, since interviewers knew who the cases were and examined cancer patients before the tumour had been removed.

Recent studies tend to agree with our present findings in showing no adverse effect of denture use (Franco et al, 1989; Kabat et al, 1989; Marshall et al, 1992; Velly et al, 1998). Although malignant transformation of denture-induced lesions has been hypothesized, histological studies of severe denture stomatitis (papillary hyperplasia) showed no evidence of epithelial dysplasias or carcinomas (Budtz-Jørgensen, 1981). As for the

*© 2000 Cancer Research Campaign*

**Table 2** Odds ratios (OR) and corresponding 95% confidence interval (CI) for cancer of the oral cavity and oro-pharynx according to oral hygiene and dentition. North-eastern Italy, 1996–1999

| | No. cases | No. controls | OR[2] | (95% CI) | OR[3] | (95% CI) |
|---|---|---|---|---|---|---|
| Self-reported: | | | | | | |
| Tooth brushing (times/day) | | | | | | |
| ≥ 2[4] | 32 | 64 | 1 | | 1 | |
| 1 | 40 | 43 | 1.8 | (1.0–3.3) | 1.1 | (0.5–2.4) |
| < 1 | 45 | 32 | 2.9 | (1.5–5.5) | 1.4 | (0.6–3.3) |
| $\chi^2_1$ for trend | | | 10.05; $P < 0.002$ | | 0.64; $P = 0.42$ | |
| Gum bleeding | | | | | | |
| Never[4] | 46 | 74 | 1 | | 1 | |
| Sometimes | 52 | 53 | 1.6 | (1.0–2.8) | 1.8 | (0.9–3.6) |
| Always/almost always | 14 | 8 | 2.7 | (1.1–7.1) | 3.9 | (1.2–12.6) |
| $\chi^2_1$ for trend | | | 5.94; $P = 0.01$ | | 6.83; $P < 0.01$ | |
| Mouthwash use (times/week) | | | | | | |
| Never[4] | 98 | 109 | 1 | | 1 | |
| 1–2 | 12 | 15 | 1.0 | (0.4–2.3) | 1.5 | (0.5–3.8) |
| > 2 | 11 | 13 | 1.0 | (0.4–2.4) | 1.2 | (0.4–3.5) |
| $\chi^2_1$ for trend | | | 0.00; $P = 0.99$ | | 0.35; $P = 0.55$ | |
| Years with dentures | | | | | | |
| Never[4] | 73 | 78 | 1 | | 1 | |
| < 10 years | 32 | 30 | 1.0 | (0.6–1.9) | 0.8 | (0.4–1.7) |
| ≥ 10 years | 27 | 40 | 0.7 | (0.4–1.3) | 0.5 | (0.2–1.2) |
| $\chi^2_1$ for trend | | | 1.15; $P = 0.28$ | | 2.25; $P = 0.13$ | |
| Dental check-ups | | | | | | |
| ≥ once every 5 years[4] | 41 | 64 | 1 | | 1 | |
| < once every 5 years | 54 | 62 | 1.4 | (0.8–2.4) | 0.8 | (0.4–1.6) |
| Never | 36 | 21 | 2.6 | (1.3–5.2) | 1.1 | (0.5–2.6) |
| $\chi^2_1$ for trend | | | 7.31; $P = 0.01$ | | 0.02; $P = 0.89$ | |
| Interviewer-reported: | | | | | | |
| Missing teeth | | | | | | |
| ≤ 5[4] | 29 | 48 | 1 | | 1 | |
| 6–15 | 32 | 42 | 1.3 | (0.7–2.6) | 1.1 | (0.5–2.6) |
| ≥ 16 | 70 | 53 | 2.5 | (1.3–4.8) | 1.4 | (0.6–3.1) |
| $\chi^2_1$ for trend | | | 7.44; $P < 0.01$ | | 0.68; $P = 0.40$ | |
| General oral condition | | | | | | |
| Good[4] | 25 | 72 | 1 | | 1 | |
| Average | 56 | 54 | 3.0 | (1.6–5.4) | 1.8 | (0.9–3.6) |
| Poor | 50 | 16 | 9.9 | (4.7–21.1) | 4.5 | (1.8–10.9) |
| $\chi^2_1$ for trend | | | 36.13; $P < 0.001$ | | 10.72; $P = 0.001$ | |

[1] Some strata do not add up to the total because of missing values. [2] Estimates from unconditional logistic regression equations, including terms for gender and age. [3] Estimates from unconditional logistic regression equations, including terms for gender, age, fruit and vegetable intake and smoking and drinking habits. [4] Reference category.

possible danger of mouthwash use, our study confirms the absence of an association (Blot et al, 1983) at least for mouthwash very low in alcohol content, such as those marketed at present (Winn et al, 1991).

Significant correlations between incidence rates of cancer of the oral cavity and those of cancers of the cervix and penis (Muñoz et al, 1990) and the report of HPV-DNA in a proportion of oral cancer biopsies (Franceschi et al, 1996) point to a possible role of sexual habits. Maden et al (1992) showed a 2.4-fold increased oral cancer risk, of borderline significance, in Washington state among men with 30 or more sexual partners compared to 4 or fewer. Ever practising oral sex showed an OR of 0.4, while herpetic infections (oral or genital), and warts (oral, genital, or common warts) were unrelated to oral cancer risk (Maden et al, 1992). Schwartz et al (1998), also from Washington state, reported that oral cancer risk increased with the number of lifetime partners (OR = 2.3 for ≥ 15 vs. 1 partner) and prior genital warts (OR = 2.2) in men, but not in women (OR = 1.0 and 0.7, respectively). Conversely, practice of oral sex did not seem to influence risk.

Maden et al (1992) found a 3-fold (excess of HPV-DNA in exfoliated buccal cells of cases compared to controls. Schwartz et al (1998) found a similar percentage of HPV-DNA in exfoliated cells of cases and controls (9%), but reported a 26% prevalence of HPV-DNA in oral cancer biopsies and an OR of 2.3 for HPV-16 capsid antibodies. The evaluation of HPV-status in cases and controls from our study is in progress (Herrero et al, 2000). The lack of influence of various indicators of sexual habits in our study does not refute per se a possible role of HPV in the aetiology of cancer of the oral cavity and oro-pharynx. It suggests, however, that either non-sexual routes of HPV transmission are involved (e.g., hand-to-mouth, Mindel and Tideman, 1999) and/or that HPV is involved only in a small proportion of oral cancers (Snijders et al, 1994; Schwartz et al, 1998).

### ACKNOWLEDGEMENTS

The authors wish to thank Dr L Barzan, Mrs O Volpato, Mrs L Zaina, Mr F Bellomo, and Mr G Di Bartolo for their collaboration in identifying and interviewing patients, and Mrs L Mei for editorial assistance. The contribution of the Italian Association for Research on Cancer, Milan, and Europe against Cancer (Grant No. SOC 96 202489 05F02) are also gratefully acknowledged.

*© 2000 Cancer Research Campaign*

**Table 3** Odds ratios (OR) and corresponding 95% confidence interval (CI)[1] for cancer of the oral cavity and oro-pharynx according to selected infectious diseases and sexual habits[2]. North-eastern Italy, 1996–1999

| | No. cases | No. controls | OR | (95% CI) |
|---|---|---|---|---|
| Warts[3] | | | | |
| Never | 108 | 117 | 1[4] | |
| Hands and feet | 19 | 24 | 1.0 | (0.5–2.3) |
| Other sites | 6 | 10 | 0.5 | (0.1–1.6) |
| Herpetic lesions[3] | | | | |
| Never | 97 | 96 | 1[4] | |
| Lip | 31 | 41 | 0.9 | (0.5–1.8) |
| Genitals and others | 5 | 17 | 0.3 | (0.1–1.0) |
| Candidiasis[3] | | | | |
| Never | 118 | 130 | 1[4] | |
| Mouth | 6 | 3 | 6.5 | (1.2–34.4) |
| Other sites | 10 | 15 | 2.0 | (0.7–5.8) |
| Lifetime no of sexual partners | | | | |
| 1 | 37 | 46 | 1[4] | |
| 2–5 | 45 | 50 | 0.6 | (0.2–1.4) |
| 6–10 | 14 | 25 | 0.5 | (0.2–1.5) |
| ≥ 11 | 28 | 19 | 1.6 | (0.5–5.1) |
| $\chi^2_1$ for trend | | | 0.71; *P* = 0.40 | |
| Lifetime no. of prostitutes[5] | | | | |
| 0 | 28 | 29 | 1[4] | |
| 1–5 | 22 | 28 | 0.9 | (0.3–2.9) |
| ≥ 6 | 18 | 13 | 1.0 | (0.3–3.4) |
| $\chi^2_1$ for trend | | | 0.01; *P* = 0.93 | |
| Practice of oral sex | | | | |
| Never | 64 | 71 | 1[4] | |
| Occasionally | 42 | 57 | 0.7 | (0.4–1.4) |
| Often/most of the time | 18 | 15 | 1.3 | (0.5–3.6) |
| $\chi^2_1$ for trend | | | 0.00; *P* = 0.98 | |
| Homosexual intercourse[5] | | | | |
| Never | 95 | 96 | 1[4] | |
| Ever | 2 | 2 | 1.0 | (0.1–13.1) |

[1] Estimates from unconditional logistic regression equations, including terms for gender and age, fruit and vegetable intake and smoking and drinking habits. [2] Some strata do not add up to the total because of missing values. [3] Infection sites are not mutually exclusive. [4] Reference category. [5] Men only.

### REFERENCES

Blot WJ, Winn DM and Fraumeni JF Jr (1983) Oral cancer and mouthwash. *J Natl Cancer Inst* **70**: 251–253

Budtz-Jørgensen E (1981) Oral mucosa lesions associated with the wearing of removable dentures. *J Oral Pathol* **10**: 65–80

Bundgaard T, Wildt J, Frydenberg M, Elbrønd O and Nielsen JE (1995) Case-control study of squamous cell cancer of the oral cavity in Denmark. *Cancer Causes Control* **6**: 57–67

Elwood JM, Pearson JCG, Skippen DH and Jackson SM (1984) Alcohol, smoking, social and occupational factors in the aetiology of cancer of the oral cavity, pharynx and larynx. *Int J Cancer* **34**: 603–612

Franceschi S, Muñoz N, Bosch XF, Snijders PJF and Walboomers JMM (1996) Human papillomavirus and cancers of the upper aerodigestive tract: a review of epidemiological and experimental evidence. *Cancer Epidemiol Biomarkers Prev* **5**: 567–575

Franceschi S, Favero A, Conti E, Talamini R, Volpe R, Negri E, Barzan L and La Vecchia C (1999) Food groups, oils and butter and cancer of the oral cavity and pharynx. *Br J Cancer* **80**: 614–620

Franco EL, Kowalski LP, Oliveira BV, Curado MP, Pereira RN, Silva ME, Fava AS and Torloni H (1989) Risk factors for oral cancer in Brazil: a case-control study. *Int J Cancer* **43**: 992–1000.

Graham S, Dayal H, Rohrer T, Swanson M, Sultz H, Shedd D and Fischman S (1977) Dentition, diet, tobacco, and alcohol in the epidemiology of oral cancer. *J Natl Cancer Inst* **59**: 1611–1618

Herrero R, Muñoz N, Franceschi S, Bosch X, Walboomers J, Meijer C, Snijders P and International Oral Cancer Study Group (2000) Abstract No 166. Proceedings of the 18th International Papillomavirus Conference, Barcelona, July 23rd–28th: 184

Kabat GC, Hebert JR and Wynder EL (1989) Risk factors for oral cancer in women. *Cancer Res* **49**: 2803–2806

Maden C, Beckmann AM, Thomas DB, McKnight B, Sherman KJ, Ashley RL, Corey L and Daling JR (1992) Human papillomaviruses, herpes simplex viruses, and the risk of oral cancer in men. *Am J Epidemiol* **135**: 1093–1102

Marshall JR, Graham S, Haughey BP, Shedd D, O'Shea R, Brasure J, Wilkinson GS and West D (1992) Smoking, alcohol, dentition and diet in the epidemiology of oral cancer. *Oral Oncol Eur J Cancer* **28B**: 9–15

Mindel A and Tideman R (1999) HPV transmission – still feeling the way. *Lancet* **354**: 2097–2098

Muñoz N, Cardis E and Teuchmann S (1990) Comparative epidemiological aspects of oro-genital cancers. In: *Papillomavirus in human pathology. Recent progress in epidermoid precancers*. Monsonego J (ed), pp. 1–12. Serono Symposia Publications from Raven Press, Volume 78, New York.

Negri E, La Vecchia C, Franceschi S and Tavani A (1993) Attributable risk of oral cancer in northern Italy. *Cancer Epidemiol Biomarkers Prev* **2**: 189–193.

Schildt E-B, Eriksson M, Hardell L and Magnuson A (1998) Oral infection and dental factors in relation to oral cancer: a Swedish case-control study. *Eur J Cancer Prev* **7**: 201–206

Schwartz SM, Daling JR, Doody DR, Wipf GC, Carter JJ, Madeleine MM, Mao E-J, Fitzgibbons ED, Huang S, Beckmann AM, McDougall JK and Galloway DA (1998) Oral cancer risk in relation to sexual history and evidence of human papillomavirus infection. *J Natl Cancer Inst* **90**: 1626–1636

Snijders PJF, Steenbergen RDM, Top B, Scott SD, Meijer CJLM and Walboomers JMM (1994) Analysis of p53 status in tonsillar carcinomas associated with human papillomavirus. *J Gen Virol* **75**: 2769–2775

Velly AM, Franco EL, Schlecht N, Pintos J, Kowalski LP, Oliverira BV and Curado MP (1998) Relationship between dental factors and risk of upper aerodigestive tract cancer. *Oral Oncol* **34**: 284–291

Winn DM, Blot WJ, McLaughlin JK, Austin DF, Greenberg AS, Preston-Martin S, Schoenberg JB and Fraumeni JF Jr (1991) Mouthwash use and oral conditions in the risk of oral and pharyngeal cancer. *Cancer Res* **51**: 3044–3047

© 2000 Cancer Research Campaign 

Wynder EL, Bross IJ and Feldman RM (1957) A study of the etiological factors in cancer of the mouth. *Cancer* **10**: 1300–1323

Zheng T, Boyle P, Hu H, Duan J, Jiang P, Ma D, Shui L, Niu S, Scully C and MacMahon B (1990) Dentition, oral hygiene, and risk of oral cancer: a case-control study in Beijing, People's Republic of China. *Cancer Causes Control* **1**: 235–241

*© 2000 Cancer Research Campaign*

